# Tumor-associated myeloid cells in cancer immunotherapy

**DOI:** 10.1186/s13045-023-01473-x

**Published:** 2023-07-06

**Authors:** Xinyu Cheng, Huilan Wang, Zhongyu Wang, Bo Zhu, Haixia Long

**Affiliations:** 1 Institute of Cancer, Xinqiao Hospital, Third Military Medical University, Chongqing, 400037 China; 2 Chongqing Key Laboratory of Immunotherapy, Chongqing, 400037 China

**Keywords:** Tumor associated myeloid cells, Myelopoiesis, Cancer immunotherapy, Immune checkpoint blockade, Tumor microenvironment, Immune evasion

## Abstract

Tumor-associated myeloid cells (TAMCs) are among the most important immune cell populations in the tumor microenvironment, and play a significant role on the efficacy of immune checkpoint blockade. Understanding the origin of TAMCs was found to be the essential to determining their functional heterogeneity and, developing cancer immunotherapy strategies. While myeloid-biased differentiation in the bone marrow has been traditionally considered as the primary source of TAMCs, the abnormal differentiation of splenic hematopoietic stem and progenitor cells, erythroid progenitor cells, and B precursor cells in the spleen, as well as embryo-derived TAMCs, have been depicted as important origins of TAMCs. This review article provides an overview of the literature with a focus on the recent research progress evaluating the heterogeneity of TAMCs origins. Moreover, this review summarizes the major therapeutic strategies targeting TAMCs with heterogeneous sources, shedding light on their implications for cancer antitumor immunotherapies.

## Introduction

Recently, immunotherapy has emerged as a new pillar of cancer treatment. Immune checkpoint blockade (ICB) therapies such as anti-PD-1/anti-PD-L1 have dramatically improved outcomes in patients with melanoma, non-small cell lung cancer, and other tumor types, making them one of the most promising therapies in the field of cancer treatment. However, despite the notable effect of these new immunotherapies on cancer treatment, a method to achieve a durable clinical response in most patients with cancer has not yet been developed. In this direction, numerous obstacles must be addressed, such as the low objective response rate, inevitable drug resistance, and limited survival benefit of patients.

Growing evidence suggests that a high infiltration of immunosuppressive cells into the tumor microenvironment (TME) correlates with poor prognosis and has a profound effect on the antitumor efficacy of ICB treatment in a variety of patients with cancer [1–3]. Tumor-associated myeloid cells (TAMCs) are the most abundant immune cells in the TME, accounting for up to 50% of the total tumor mass in solid tumors. Generally, TAMCs are mainly represented by two populations: tumor-associated macrophages (TAMs) and myeloid-derived suppressor cells (MDSCs), which promote tumor growth either directly by favoring tumor cell proliferation and survival or indirectly by creating an immunosuppressive microenvironment [4–6]. Most importantly, given their abundance and strong T-cell suppression potency, a large body of evidence has shown that TAMCs exert profound effects on resistance to ICB treatment. TAMC infiltration is closely correlated with CD8^+^ T-cell exhaustion and negatively correlated with ICB efficacy [7]. The dual inhibition of TAMs and granulocytic MDSCs can significantly enhance the efficacy of ICB [8]. Although combination immunotherapy strategies targeting TAMCs have been widely developed, the results have been modest. Hence, it is crucial to understand the characteristics of TAMCs.

A major challenge in developing TAMCs target strategies is that TAMCs are highly heterogeneous in their functions and origins. Recently, a more holistic analysis of TAMCs using single-cell RNA sequencing revealed the diversity of TAMCs in a wide range of cancers, including lung, breast, and ovarian cancers [9–12]. Recent studies have revealed significant functional differences among TAMCs derived from different sources, indicating that TAMCs' origin is the key to determining functional heterogeneity. In addition to the classical origin of bone marrow (BM), the spleen and embryo have recently emerged as non-negligible sites for TAMCs production. The abnormal differentiation of hematopoietic stem and progenitor cells (HSPCs), erythroid progenitor cells, B precursor cells, and embryo-derived TAMCs are also an important origins of TAMCs. These myeloid cells exhibiting origin heterogeneity have unique phenotypes and functional characteristics. Moreover, they acquire multiple mechanisms to impair the efficacy of ICB (Table 1). Understanding the origin and functional heterogeneity of TAMCs is important for developing precisely targeted intervention strategies, providing new ideas for overcoming myeloid immunosuppression, restoring protective T-cell immunity, and enhancing the efficacy of ICB synergistic therapy in patients with cancer.

**Table 1:** The characteristics of heterogeneous TAMC

Cell type	Origin	Biomarker	Regulatory Mechanism	Immunophenotype	Function Features
TAM	Monocytic precursors in bone marrow, spleen and embryo	M1: F4/80^+^CD11b^+^CD86^+^ M2: F4/80^+^CD11b^+^CD206^+^ Embryo-specific: CX3CR1^high^CD11a^low^ CD49d^low^	(a) Differentiation: CSF-1, TGF-β, VEGF, platelet-derived growth factor (b) Polarization: M1 (IFN-γ, lipopolysaccharide); M2 (IL-4, IL-13) (c) Proliferation: GM-CSF and adenosine Embryo-specific: (a) AngII-AGTR1A increase HSCs retention (b) EMPs buds from the yolk sac endothelium (c) Colonize the embryonic tissue or liver (d) Differentiate from the early or later wave	M1: iNOS, ROS, IL-1β, TNF-α, IL-12, IFN-γ M2: ARG1, IL-10, TGF-β, VEGF, MMP	M1: (a) Kill tumor cells (b) Activate NK cells and cytotoxic T cells M2: (a) Inhibit T cells and NK cells activition (b) Induce angiogenesis (c) Promote tumor growth and invasion (d) Recruit Tregs into TME Embryo-specific: Pro-fibrotic
MDSC	M-MDSC: Monocytic precursors in bone marrow and spleen PMN-MDSC: Granulocytic precursors, monocytic-like precursors in Bone marrow and spleen	M-MDSC: CD11b^+^CD14^+^HLA-DR^–/lo^CD15^−^ (human), CD11b^+^Ly6G^−^Ly6C^hi^ (mouse) PMN-MDSC: CD11b^+^CD14^−^CD15^+^/CD66b^+^ (human), CD11b^+^Ly6G^+^Ly6C^lo^ (mouse) Splenic MDSC: PMN-like cell	(a) Differentiation: IL-1β, IL-6, S100A8-9, IFN-γ, IL-4, IL-13, IL-10 (b) Accumulation and expansion: IL-6, IL-10, TGF-β Splenic MDSC-specific: (a) Recruit specific HSPCs via CCL2/CCR2 (b) Endogenous GM-CSF signals and local education by the splenic stroma	M-MDSC: iNOS, ARG1, IL-10, TGF-β PMN-MDSC: ROS, PGE2, IDO, ARG1	(a) Reduce the anti-tumor activity of T cells (b) Inhibit NK cells, macrophages, dendritic cells function (c) Induce Tregs
TAN	Granulocytic precursors in Bone marrow	CD15^+^CD16^+^CD66b^+^CD14^−^ (human), CD11b^+^Ly6G^+^ (mouse)	Polarization: N1 (type 1 interferon); N2 (TGF-β)	CCL2, CCL17	N1: Kill tumor cells directly Activate dendritic cells, CD4^+^ T cells, etc N2: Inhibit tumor cell apoptosis Promote angiogenesis Inhibit CD8^+^ T cells function Promote tumor invasion and metastasis Recruite macrophages and Tregs into TME
TADC	Dendritic cell precursors in bone marrow	pDC: BDCA-2^+^BDCA-4^+^IL-3Ra^+^ CD11c^−^ (human), CD45R^+^CD317^+^Siglec-H^+^ CD11c^low^ (mouse) cDC1: BDCA3^+^CD141^high^ cDC2: BDCA1^+^CD1c^+^ moDC: Indistinguishable from cDC2	Development and maintenance of phenotype: (a) pDCs: E protein transcription factor E2-2 (b) cDCs: E protein antagonist Id2	pDC: IDO, ICOSL, PD-L1, granulosase B cDC1: TNF-α, IL-6, IL-8, IL-12, CXCL9, CXCL10 cDC2: IL-1β, IL-6, IL-12, IL-23 moDC: NOS2, CD40 L, TNF	pDC: Inhibit CD8^+^ T cells function Induce Tregs cDC1: Cross-presentation Polarize and activate CD4^+^ T cells Activate NK cell Recruit CD8^+^ T cells into TME cDC2: Activate CD4^+^ T cells moDC: Promote CD8^+^ T cell to kill tumor cells
EDMC	CD45^+^ EPCs in extramedullary organ (especially spleen)	CD235a^+^CD71^+^CD11b^+^CD33^+^HLA-DR^−^ (human), Ter119^+^CD71^+^CD11b^+^Gr1^+^ (mouse)	(a) Tumors block the default erythrocyte differentiation pathway of CD45^+^ EPCs (b) GM-CSF… mediate erythrocyte-myeloid trans-differentiation	High level of PD-L1, Arg-1, iNos, ROS, et al	Strong ability to inhibit CD8^+^T cell proliferation and IFN-γ production, promote tumor growth and invasion
B-MF	Pre-B in local tumor	CD19^+^CD79a^+^IgM^+^ TAM	(a) Tumors mobilize BM pre-B accumulation in the spleen (b) Acquisition of myeloid characteristics: Tumors decrease PAX5 levels using M-CSF	High level of PD-L2, B7-H3, Marco, TGF-β, et al	(a) Inhibit CD4^+^ T cell and induce Treg (b) Unique metabolic and inflammatory functions (c) Strong phagocytic ability

In this review, we summarize the critical progress in understanding the origin of TAMCs via tumor-trained myelogenesis and the role of these heterogeneous TAMCs in inducing immune evasion and immunotherapeutic resistance. Finally, we propose prospective targeting strategies that could be exploited to enhance anticancer immunotherapy.

## Origin of classical TAMCs

### Tumor-trained myeloid-biased differentiation in BM

**Fig. 1:**
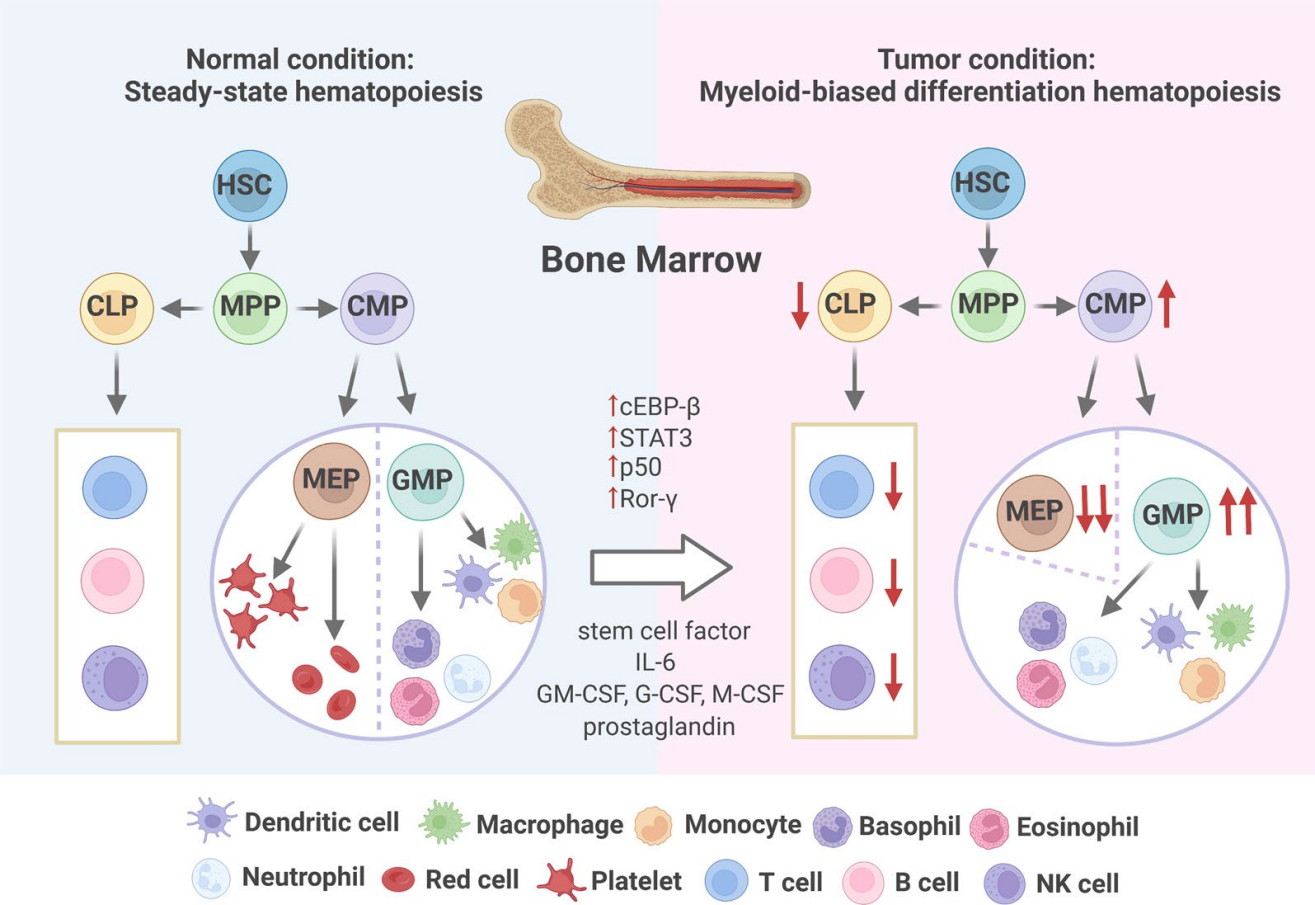
The origin and regulatory mechanism of classical TAMCs. Normally, there is a steady-state myelopoiesis in the bone marrow, producting mature erythrocytes, lymphocytes, and myeloid cells. However, tumors induce myeloid-biased differentiation via upregulation of some transcription factors and action of various regulatory factors. On the basis of downregulating CLP ratio, GMP level increases significantly and immature myeloid cells accumulate in the bone marrow

Steady-state hematopoiesis in the BM is a strictly regulated process of step-by-step lineage commitment that ensures the continuous generation of all blood lineages. Conventionally, myelopoiesis is initiated at the first branching point when an HSPC makes the binary choice to become either a common myeloid progenitor (CMP) or a common lymphoid progenitor (CLP). CMPs lose their differentiation potential after commitment to granulocyte–macrophage progenitors (GMPs) or megakaryocyte-erythroid progenitors (MEPs). GMPs are known to differentiate into neutrophils, macrophages, and MDSCs. As for MEPs, they simultaneously produce megakaryocytes and erythrocytes [13]. However, in carcinogenesis, steady-state hematopoiesis is perturbed and is characterized by the preferential differentiation of myeloid cells at the expense of erythrocytes and lymphocytes [14–16] **(**Fig. 1**)**.

Tumor-trained myeloid-biased differentiation of HSPC has been widely reported. A four- to seven-fold increase in the levels of circulating GMPs was observed in 133 patients with seven different types of tumors, exhibiting a generalized myeloid bias of HSPCs with a skew toward granulocytic differentiation. The main mechanisms of myeloid bias include impaired differentiation into mature myeloid cells and targeted reprogramming of myeloid differentiation from an early hematopoietic compartment, which results in the accumulation and persistence of immature myeloid cells [14, 17, 18]. Specifically, a regulatory network including stem cell factor, granulocyte–macrophage colony-stimulating factor (GM-CSF), IL-6, and prostaglandins drives the expansion of heterogeneous immature myeloid cells and blocks their maturation [19–22]. In addition, the upregulation of transcription factors, such as cEBP-β, STAT3, p50, and Ror-β, also contributes to myeloid-biased differentiation. Clinical data have also confirmed that in colorectal cancer and hepatocellular carcinoma (HCC), myeloid-biased differentiation, such as an elevated preoperative neutrophil-to-lymphocyte ratio, is a poor prognostic predictor of colorectal cancer and HCC [23, 24].

Macrophages, MDSCs, neutrophils, and dendritic cells derived from tumor-trained myeloid-biased differentiation migrate to local tumors under the action of different chemokines and perform specific biological functions (Fig. 4).

### Tumor-associated macrophage (TAM)

#### M1-TAM and M2-TAM

Several molecules such as CCL2, CCL5, colony-stimulating factor 1 (CSF-1), transforming growth factor-beta (TGF-β), vascular endothelial growth factor (VEGF), and platelet-derived growth factor can recruit monocytes into TME and promote their differentiation into TAM [25, 26]. Macrophages are classified into two categories based on their surface receptor expression and secreted products: classically activated macrophages (M1) polarized by IFN-γ and lipopolysaccharide, and alternatively activated macrophages (M2) polarized by IL-4 and IL-13 [27]. Interestingly, the polarization state of TAM is plastic. High levels of proliferator receptor gamma (PPARγ), enhancement of the NF-κB signaling pathway, and a weakly acid or hypoxic TME promote the polarization of TAM from the M1 phenotype to the M2 phenotype. When these conditions are suppressed, M2 can be repolarized into M1 [28]. Functionally, M1 (F4/80^+^CD11b^+^CD86^+^) can kill tumor cells by expressing inducible nitric oxide synthase (iNOS), reactive oxygen species (ROS), and cytokines such as IL-1β, tumor necrosis factor-alpha (TNF-α), IL-12 and IFN-γ. Moreover, M1 also triggers the activity of natural killer (NK) cells and prime cytotoxic T-cells. Conversely, M2 (F4/80^+^CD11b^+^CD206^+^) inhibits the activity of T-cells and NK cells by expressing ARG-1, IL-10, and TGF-β, induces angiogenesis and promotes tumor growth and invasion by expressing VEGF and matrix metal protein (MMP), and can also generate chemokines such as CCL2 and CCL5 to recruit regulatory T-cells (Tregs). In the TME, the phenotypic change of TAM is characterized by a significant increase in the proportion of M2 with tumor progression, which is associated with poor prognosis in many malignancies [29]. M2-TAMs have been shown to be largely responsible for PD-1/PD-L1 blockade resistance by inducing T-cell exclusion within tumor sites and secreting indoleamine 2, 3-dioxygenase (IDO) to inhibit T-cell functions [30]. However, increasing evidence indicates that there are many macrophages with overlapping M1 and M2 characteristics between pro-inflammatory M1 and anti-inflammatory M2 cells [31]. Therefore, the heterogeneity of TAM in the TME is more complex than what we previously thought of as a binary classification. For example, Peng et al. used scRNA-seq to redefine five TAM subtypes based on gene expression profiles rather than on phenotypes. Genes involved in Class II antigen presentation (*HLA* and *CD74*), genes related to extracellular matrix (ECM) deposition and remodeling (*COL1A*), genes for the recruitment of myeloid cells (*CCL2*), chemokine genes responsible for cytotoxic T-cell infiltration (*CXCL9 and CXCL10*), and genes that generate pro-inflammatory cytokines (*S100A8 and S100A9*) were highly expressed in the five cell clusters [32].

#### Proliferating TAM in local tumors

Emerging evidence indicates that after site transition from the periphery to the TME, TAMs can still accumulate through tumor-trained proliferation. Recent studies using mouse models have indicated that the proliferative capacity of TAMs is higher than that of mammary tissue macrophages in breast cancer tissues, promoting tumor growth [33]. Furthermore, analysis of clinical liver cancer tissues has revealed a high level of TAM proliferation [34]. However, the precise mechanisms that regulate TAM self-renewal remain unclear.

Wang et al. found that adenosine, produced by adenosine monophosphate (AMP) and catalyzed by CD73 in hepatoma cells, is an essential element in the induction of TAM proliferation. Simultaneously, autocrine GM-CSF released by tumor-stimulated TAMs enhances A2A receptor expression in TAMs. Moreover, they demonstrated that GM-CSF functions synergistically with adenosine to activate the downstream AKT/ERK signaling pathway, inducing TAM proliferation and promoting tumor progression. Therefore, their findings further support the hypothesis that TAMs exhibit increased proliferation, which is an important mechanism contributing to TAMs accumulation in human HCC. These self-replicating TAMs (Ki67^+^ TAM) exhibit an immunosuppressive phenotype and are inversely correlated with the density of CD8^+^ T cells, playing a role in promoting tumors and predicting a poor prognosis. Kaplan–Meier analysis showed that local TAM replication was correlated with reduced overall survival (OS) and recurrence time in patients with HCC [34]. Expression levels of the adenosine receptor ADORA2B were negatively correlated with the OS of patients with HCC [35]. Therefore, through this mechanism, self-proliferation may be a potential target for future antitumor immunotherapy, providing a new strategy for inhibiting TAM accumulation in HCC.

### MDSC

Chemokines such as CCL2, CXCL5, and CXCL8 recruit immature myeloid cells to the tumor stroma [36]. Immature myeloid cells become immunosuppressive MDSC under the combined influence of tumor-derived IL-1β, IL-6, and S100A8-9, as well as IFN-β, IL-4, IL-13, and IL-10 released by activated T cells [37]. Cytokines, such as IL-6, IL-10, and TGF-β, promote the accumulation and expansion of MDSC by activating the JAK/STAT1 and JAK/STAT3 signaling pathways [38].

MDSCs are divided into two main subpopulations based on their direction of differentiation: monocytic (M-MDSCs) and polymorphonuclear (PMN-MDSCs). In mice, M-MDSCs were defined as CD11b^+^Ly6G^−^Ly6C^hi^, whereas PMN-MDSCs were defined as CD11b^+^Ly6G^+^Ly6C^lo^. In humans, M-MDSCs are CD11b^+^CD14^+^HLA-DR^–/lo^CD15^−^, whereas PMN-MDSCs are CD11b^+^CD14^−^CD15^+^/CD66b^+^. Although M-MDSCs and PMN-MDSCs appear to develop along divergent pathways, studies have shown that histone deacetylase 2 (HDAC-2) can mediate transcriptional silencing of the retinoblastoma gene in myeloid cells to enable M-MDSCs to acquire the PMN-MDSC phenotype, which is an important reason for PMN-MDSC accumulation in patients with tumors [39]. Upon entry into the TME, MDSCs impair the antitumor activity of T cells; inhibit the function of NK cells, macrophages, and dendritic cells; and induce Tregs by producing ARG-1, iNOS, TGF-β, IL-10, cyclooxygenase-2 (COX-2), and IDO [40]. Accumulating evidence demonstrates that high MDSC infiltration is negatively associated with the efficacy of ICBs in multiple tumor types. Therefore, therapeutic strategies targeting MDSC in combination with ICBs have been extensively tested [41].

### Tumor-associated neutrophil (TAN)

The CXCL2-CXCR2 axis and cytokines such as GM-CSF, TNF-α, and IFN-γ attract neutrophils into TME, where neutrophils are trained into TANs. In mice, TANs are defined as CD11b^+^Ly6G^+^, while in humans, they are defined as CD15^+^CD16^+^CD66b^+^CD14^−^. Similar to macrophages, TANs are classified into a functionally distinct antitumorigenic phenotype (N1) and a protumorigenic phenotype (N2). Studies have shown that TGF-β alters neutrophilic phenotype into N2, while type 1 interferon alters neutrophilic phenotype into N1 [42, 43]. The key mediator of N2 carcinogenesis is MMP-9, which plays an important role in inhibiting tumor cell apoptosis and promoting angiogenesis. Additionally, N2 inhibits the function of CD8^+^ T cells, contributes to tumor invasion and metastasis, and promotes tumor progression by recruiting macrophages and Tregs via CCL2 and CCL17. N1 plays an antitumor role by killing tumor cells directly or by activating dendritic cells, CD4^+^ T cells, and other immune cells [44]. Although the TME polarizes TAN to N2, angiotensin-converting enzyme inhibitors (ACEi) and angiotensin II type 1 receptor (AGTR1) antagonists can polarize it toward N1 to attenuate tumor growth [45]. Studies have confirmed that higher TAN densities after ICB treatment result in poorer OS [46]. Moreover, targeted TAN therapy has been reported to improve the efficacy of ICB therapy. However, accurate targeting of N2 without destroying N1 to protect normal immune function remains a critical challenge [47].

### Tumor-associated dendritic cell (TADC)

TADC populations are composed of three major subgroups: plasmacytoid DC (pDC), conventional DC (cDC1 and cDC2), and monocyte-derived DC (moDC) [48]. Tumors recruit pDCs from lymphatic organs to the TME by secreting CXCL12. pDCs were identified as BDCA-2^+^BDCA-4^+^IL-3Ra^+^CD11c^−^ in humans and CD45R^+^CD317^+^Siglec-H^+^CD11c^low^ in mice. pDCs express high levels of IDO, ICOSL, and PD-L1. Moreover, they produce granulosase B to inhibit CD8^+^ T cells and induce Tregs, thus promoting tumor progression.

In contrast, cDCs play an antitumor role. cDCs migrate from the peripheral tissue to the TME under the action of the chemokines CCL5 and XCL1 and are mainly divided into cDC1s (BDCA3^+^CD141^high^) and cDC2s (BDCA1^+^CD1c^+^). cDC1 is the main DC subgroup that migrates to lymph nodes for cross-presentation. At the same time, cDC1 also produces inflammatory cytokines such as TNF-α, IL-6, IL-8, and IL-12 to promote CD4^+^ T cell polarization and NK cell activation and generates CXCL9 and CXCL10 to recruit CD8^+^ T cells into TME. cDC2 produces various cytokines, such as IL-1β, IL-6, IL-12, and IL-23, to activate CD4^+^ T cells.

MoDCs are derived from monocytes and have a phenotype similar to cDC2, making them difficult to distinguish. The expression of NOS2, CD40 L, and TNF in MoDCs is closely related to the destructive effects of CD8^+^ T cells in the TME [49].

TADCs, which are rich in PD-L1 expression, are an important target of PD-L1 blocking antibodies. Therefore, ICBs can reinvigorate DC function to generate effective antitumor T-cell immunity [50]. Notably, studies using scRNA-seq analysis have further clustered this activated DC population as cDC1 [51].

Next, we summarized non-BM-derived TAMCs, which are critical components of TAMC heterogeneity that may have been overlooked in previous studies.

## Splenic HSPCs mediated tumor-promoting myelopoiesis

Myeloid cells are usually short-lived and require constant replenishment by HSPCs as cancer progresses [52]. In addition to the BM, HSPCs exist in the spleen and play an important role in supplying myeloid cells to promote tumor progression. Previously, the spleen was considered an important extramedullary reservoir of myeloid cells, specifically monocytes, which are fundamentally important for tumor-induced tolerance. However, a growing body of research has shown that tumor-trained myeloid-biased differentiation also occurs in the spleen, which is the main lymphoid organ that undergoes myeloid cell expansion during tumor development. There is strong evidence that splenectomy can significantly decrease the number of TAMCs, restore protective T-cell immunity, and induce tumor regression when synergized with ICB. Splenic CD11b^+^Gr-1^int^Ly6C^hi^ cells with myeloid progenitor features expand in the marginal zone of the spleen, induce the cross-presentation of tumor antigens, and cause CD8^+^ T cell tolerance [53].

**Fig. 2:**
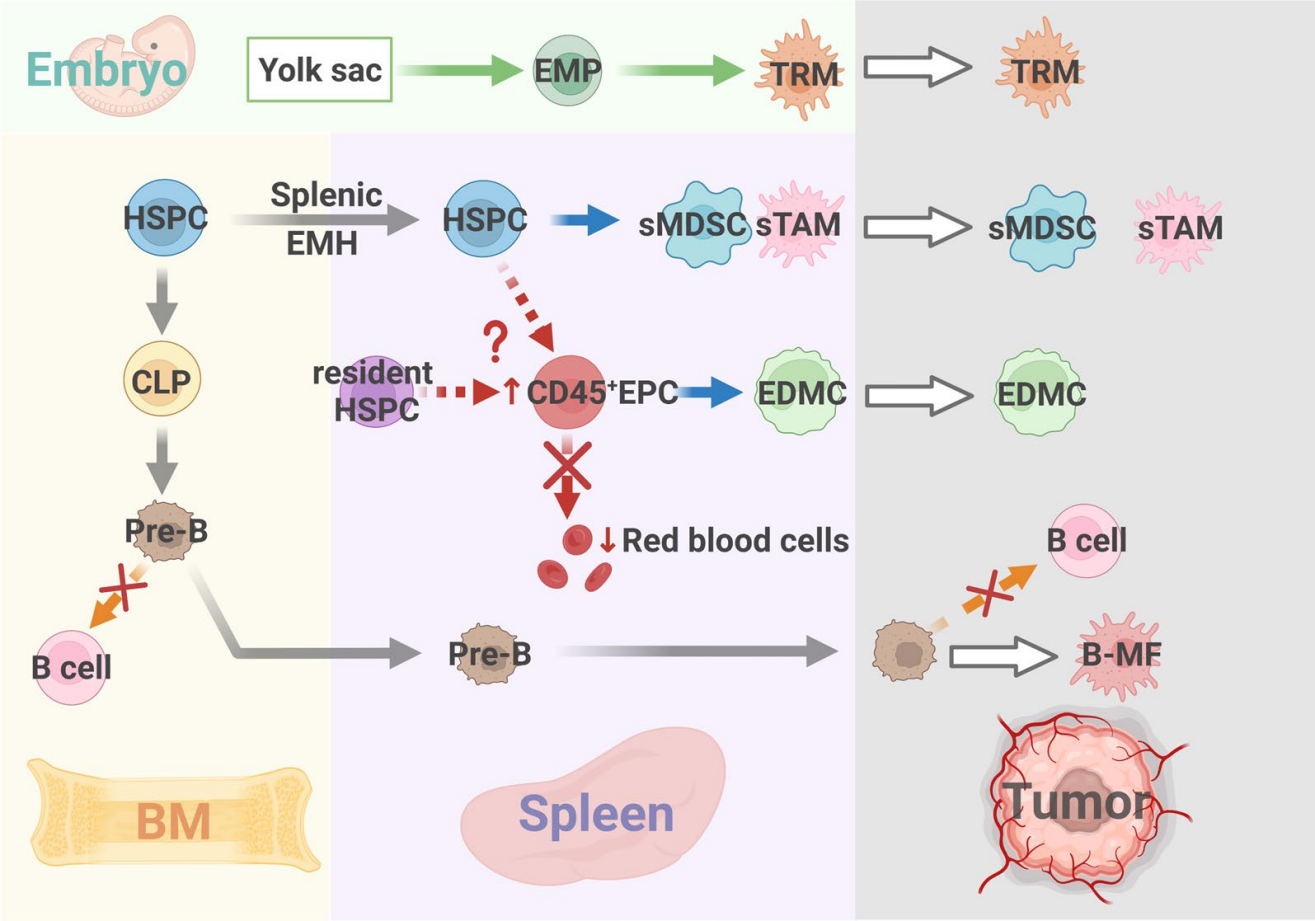
The origin of non-classical TAMCs. BM, spleen and embryo are the main sites of TAMCs differentiation, and the local tumor is the functional site of TAMCs. (1) TRMs constitute embryo-derived TAM. TRMs are derived from EMPs, which form in the yolk sac during embryogenesis. (2) Tumors induce splenic EMH, generating splenic MDSC & TAM (sMDSC, sTAM). (3) CD45^+ ^EPCs accumulate in spleen. The red blood cell differentiation pathway of CD45^+ ^EPC is blocked by tumors, thus it trans-differentiates into EDMC. (4) Pre-B doesn’t differentiate into B cell, but migrates from BM to the spleen and eventually trans-differentiates into B-MF in the tumor microenvironment

**Fig. 3:**
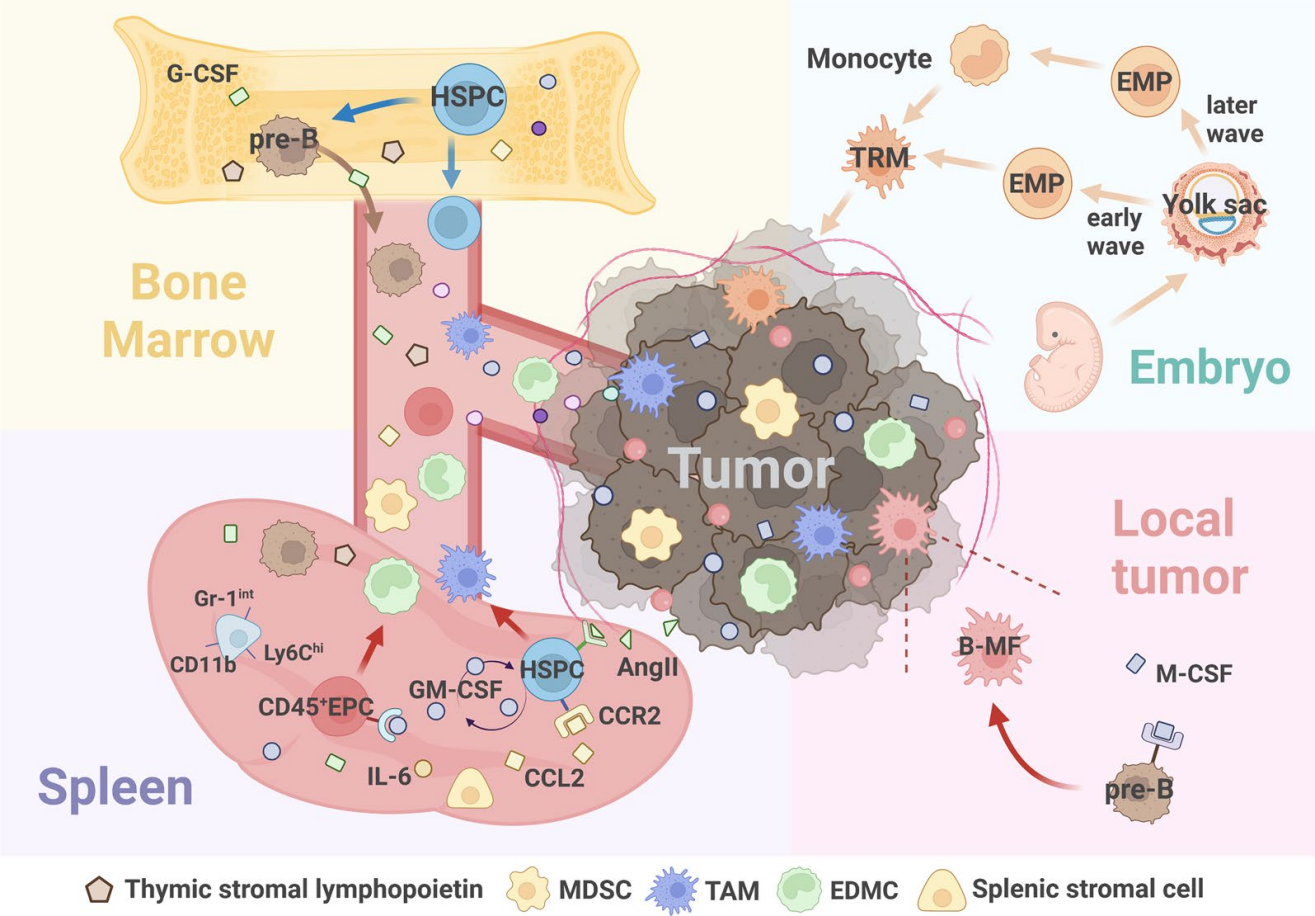
The regulatory mechanism of non-classical TAMCs. (1) Spleen EMH: Splenic CD11b^+^Gr-1^int^Ly6C^hi^ cells expand in the marginal zone of the spleen. Spleen recruits specific HSPCs subpopulation from BM via the CCL2/CCR2 axis, and HSPC differentiates into TAMCs driven by endogenous GM-CSF signals and splenic local education. The AngII-AGTR1A signaling pathway increases the retention of HSPCs in the spleen. (2) Tumors block the default red blood cell differentiation pathway of CD45^+ ^EPCs in the spleen. Under the effect of GM-CSF, CD45^+ ^EPCs trans-differentiate into EDMCs. (3) During embryogenesis, EMPs are formed in the yolk sac. TRMs are derived from both early wave and late wave EMPs. (4) Cancers use thymic stromal lymphopoietin and G-CSF to mobilize BM pre-B accumulation in the spleen. In tumor microenvironment, pre-B responds to cancer-secreted M-CSF and trans-differentiates into B-MF

In recent years, extramedullary hematopoiesis (EMH) in the spleen, which is important for the production of myeloid suppressor cells in the tumor state, has received extensive attention. With the increasing demand for hematopoiesis and damage to the hematopoietic function of the BM, the tumor-bearing host activates extramedullary organs, particularly the spleen, to supplement hematopoietic function. Heightened splenic myelopoiesis in cancer can be linked to a large accumulation of HSCs and GMPs within the splenic red pulp of tumor-bearing hosts. These progenitor cells locally produce monocytes and granulocytes [53]. Researchers have revealed that splenic EMH is not only a quantitative supplement to BM hematopoiesis in tumor-bearing hosts but also has a unique and important function in generating immunosuppressive myeloid cells [54]. They found that the splenic stromal cells of tumor-bearing mice recruited specific subsets of HSPCs into peripheral circulation via the CCL2/CCR2 axis. Endogenous GM-CSF signaling and local education through the splenic stroma, such as the production of IL-6, synergistically drive the myeloid commitment of HSPCs to differentiate into potent immunosuppressive myeloid cells. Therefore, the selective recruitment of circulating HSPCs and splenic niche signals is an integral and essential process that promotes the systemic myeloid response (Fig. 2). Moreover, Cortez-Retamozo et al. [55] discovered a special mechanism for the retention of this particular group of HSPCs in the spleen, which strongly proved that this extramedullary source could sustain a sufficient supply of TAMCs. They found that a fraction of the angiotensinogen produced by tumors may be locally converted into Ang II in a mouse model of lung adenocarcinoma. AngII depends on AngII-AGTR1A signal transduction in hematopoietic cells to suppress the signaling between sphingosine-1-phosphate receptor 1 and sphingosine-1-phosphate and significantly increases the retention of HSCs in the spleen, thus amplifying the macrophage progenitor response in vivo and consequently facilitating the supply of TAM in the spleen during cancer progression. Therefore, the reprogramming of AngII-dependent HSPC trafficking expanded splenic HSPCs in tumor-bearing mice and increased the number of TAM in the tumor tissue at its source (Fig. 3).

Although circulating HSPCs are an important source of splenic HSPCs, splenic HSPCs are characterized by their ability to support stress-induced myelogenesis. Splenic HSPCs include primed progenitors of potent myeloid suppressors that readily respond to endogenous GM-CSF signals and produce highly inhibited myeloid descendants. Thus, they can directly inhibit the proliferation and antigen-induced cytotoxic activity of anti-CD3 and anti-CD28 stimulated T cells, regardless of the presence of tumors. Splenic HSPCs are considered negative clinical indicators, and a higher frequency of CD133 expression is associated with poorer patient prognosis [54]. As tumor-generated angiotensinogen is indicative of splenic HSPCs retention, the angiotensinogen level in human patients is associated with clinical prognosis and has reference value [55].

Collectively, splenic HSPCs are critical for the generation of TAMCs and the impairment of antitumor immunity, providing promising antitumor immunotherapy strategies to restrain systemic tumor-promoting myelopoiesis at its source.

## Tumor-induced erythroid precursor-differentiated myeloid cells

Previous studies on TAMCs have mainly focused on the leukocyte immune system; however, the role of erythroid cells in tumor immunity has been neglected. Evidence suggests that erythroid cells have both immunosuppressive and immunomodulatory properties [56–59]. In addition to splenic HSPCs, erythroid progenitor cells in the spleen are directly involved in the generation.

Abnormal tumor-trained extramedullary erythropoiesis leads to the accumulation of a large number of erythroid precursor cells (EPCs) in extramedullary hematopoietic organs, specifically the spleen [60]. As robust immunosuppressors, the CD45 subpopulation of EPCs behaves similarly to MDSC-like cells, causing systemic impairment of CD8^+^ T cell-mediated immune responses [61]. Recently, Long et al. showed that the traditional hematopoietic development model was disrupted under tumor regulation and that tumors blocked the default red blood cell differentiation pathway of CD45^+^ EPCs (Fig. 2). Under the effect of GM-CSF, they transform into myeloid cells, becoming an important origin of TAMCs in the TME, revealing a new production mechanism for TAMCs [62]. This cellular "erythroid-myeloid hybrid" population carries both erythroid and myeloid-specific markers, named "erythroid-differentiated myeloid cells (EDMCs)" (Fig. 3). Moreover, this process creates a feed-forward mechanism whereby sustained anemia repeatedly triggers extramedullary erythropoiesis. However, the tumor diverts extramedullary erythropoiesis from red blood cells to EDMCs, ultimately resulting in the continuous production of erythroid-derived TAMCs.

**Fig. 4:**
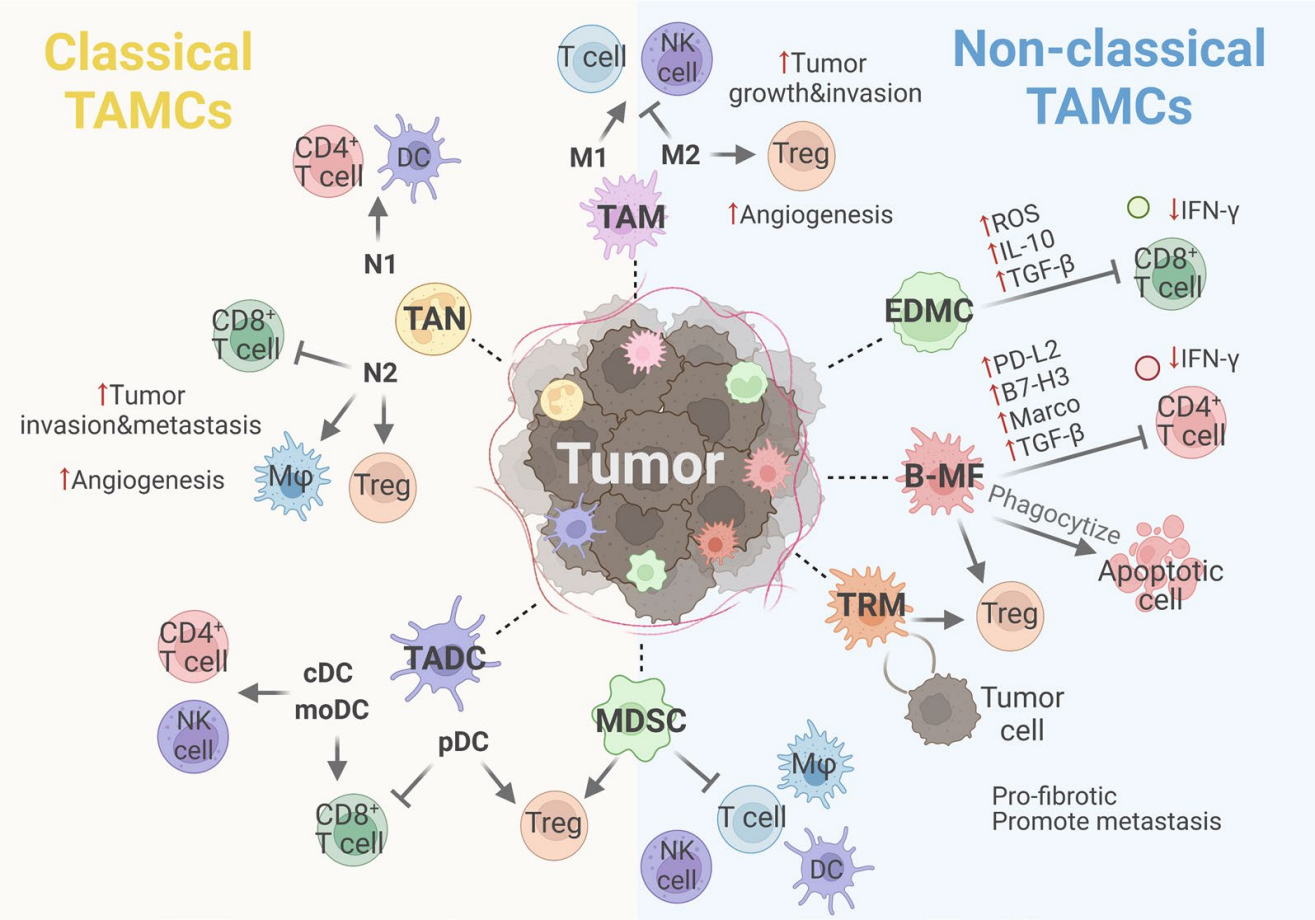
Functional characteristics of heterogeneous TAMCs. (1) Bone marrow and spleen derived TAM includes anti-tumor M1 and pro-tumor M2. (2) TAN includes anti-tumor N1 and pro-tumor N2. (3) TADC populations mainly composed of three major subgroups, including pDC, cDC and moDC. (4) Bone marrow and spleen derived MDSC can not only reduce the anti-tumor activity of T cells, but also inhibit the function of NK cells, macrophages and dendritic cells, as well as induce Tregs. (5) EDMC shows strong immunosuppressive function by producing ROS, IL-10, TGF-β and inhibiting T cell production of IFN-γ. (6) B-MF significantly inhibits CD4^+^ T cells proliferation and IFN-γ production, phagocytizes apoptotic cells, and more effectively induces the production of Treg. (7) TRM induces Treg response, and also shows obvious pro-fibrotic transcriptional profile and has a causative role in tumor metastasis and spread. And there are spatial interactions of TRMs with tumor cells. An indication with an arrow indicates activation, induction, recruitment, apoptosisinhibition, cross-presentation, etc., while an indication without an arrow indicates inhibition, killing, etc.

Functionally, EDMCs are more effective in suppressing CD8^+^ T-cell proliferation and IFN-γ production by expressing high levels of PD-L1, ARG-1, and NOS2. Although both classical MDSCs and EDMCs accelerated tumor growth and attenuated the efficacy of anti-PD-L1 treatment, EDMCs mediated stronger suppression than classical MDSCs (Fig. 4). Clinically, the number of intratumoral EDMCs predicts T-cell exhaustion and a tolerant microenvironment in the majority of tumor types. Additionally, high infiltration of EDMCs was negatively correlated with the therapeutic efficacy of the PD-1/PD-L1 antibody in patients with cancer, and its predictive ability was significantly better than that of traditional immunosuppressive cells such as Tregs and MDSCs. Moreover, a large number of CD45^+^CD71^+^ erythroid cells, consistent with EDMCs in all features, were found in the HCC tissues, although no definite detection of myeloid markers has been reported. These cells exhibited stronger suppressive functions than MDSCs by producing ROS, IL-10, and TGF-β, and the degree of infiltration predicted disease-free survival and OS; its prognostic value was better than that of the Cancer of the Liver Italian Program score, which meant it may be a new clinical method for predicting HCC tumor recurrence [63]. Because extramedullary erythropoiesis and EDMCs production are repeatedly triggered by anemia, anemia has also been proven to be an effective indicator for predicting the efficacy of clinical immunotherapy, which provides strong guidance for screening the dominant population of immunotherapy and lays a foundation for the formulation of combination therapy strategies [62].

In conclusion, the lineage switching of EPCs caused by tumor-trained extramedullary erythropoiesis is an important origin of TAMCs. The above-mentioned studies provide new ideas for developing combined immunotherapy strategies targeting TAMCs and may provide an effective combinational strategy to improve ICB therapeutic efficacy in the future.

## Tumor-induced B precursor cells differentiated macrophage-like cells

Similar to erythroid-myeloid transdifferentiation, B-lymphocyte-derived TAMs also challenge the traditional HSPC differentiation model by adding a new cell-derived feature to the heterogeneity and plasticity of TAMCs. Studies suggest that under pathological conditions such as inflammation and tumors, B-cell precursors (pre-B) appear to retain macrophage differentiation potential as an additional source of macrophages in vivo. Audzevich et al. [64] found that, under the influence of the tissue environment and inflammatory signals, macrophage precursors with myeloid transdifferentiation potential and functional plasticity exist within the BM early pro-B cell compartment, and they co-express myeloid (GR1, CD11b, and CD16/32) and lymphoid (B220 and CD19) lineage markers. Moreover, cancer growth at distant sites can remotely affect BM hematopoiesis by inhibiting B-cell lymphopoiesis and skewing the differentiation of precursor cells toward myelopoiesis [65].

In addition, a growing body of research has reported an increase in the total number of splenic B cells and an elevated frequency of marginal zone B cells, which depend on tumor burden. Chen et al. [66] recently reported that tumors use thymic stromal lymphopoietin and granulocyte colony-stimulating factor (G-CSF) to mobilize BM pre-B accumulation in the spleen (Fig. 2). These cells responded to cancer-secreted macrophage colony-stimulating factor (M-CSF) with downregulation of the transcription factor Pax5 via CSF1R signaling and eventually trans-differentiated into TAM (pre-B generate macrophage-like cells, termed "B-MF”) in TME (Fig. 3). These cells express markers such as CD19, CD79a, and IgM. This pre-B cell transdifferentiation pathway is not unique to mice because B-MF-like cells and their transcriptional characteristics have also been detected in patients with cancer.

Consistent with significant upregulation of genes associated with phagocytosis, M2-skewing, and immunosuppressive functions (PD-L2, B7-H3, Marco, TGF-β), B-MF negatively regulated antitumor IFNγ^+^CD4^+^ T cells. Compared to monocyte-derived macrophages, B-MFs more efficiently induced the generation of Tregs and increased cancer progression and metastasis. B-MFs showed unique metabolic and inflammatory functions and efficiently phagocytosed apoptotic cells. This suggests that the cancer-induced pre-B cell transdifferentiation pathway is biologically functional (Fig. 4).

Biphenotypic pre-B with macrophage differentiation potential is widely present in various tumors, which has significantly advanced our understanding of heterogeneity [11, 67]. The discovery of this origin provides new insights into the role of B cells in tumor progression. To date, B cells have not been classified as good or bad indicators of cancer. Even within the same tumor model, different types of B cells can show different abilities to promote or delay tumor escape. This subgroup, which can differentiate into TAMCs, may be an important component of the B cells that exert immunosuppressive activity.

## Embryo-derived TAM

The aforementioned studies represent the main emerging findings in recent years regarding the non-classical origin of TAMCs. Notably, embryo-derived TAMs are the earliest and most widely accepted specific origin and have been confirmed in many studies (Fig. 2). This finding reverses the traditional belief that all tissue macrophages are derived from HSPCs in the BM. Normally, tissue-resident macrophages (TRMs) remain in situ and receive some degree of replenishment from circulating monocytes throughout adulthood [68]. Studies have shown that they can be divided into two categories depending on when and how they are produced. The early wave of erythro-myeloid progenitor (EMPs) buds from the yolk sac endothelium between E7.0 and E8.25, differentiates into yolk sac macrophages in the absence of monocyte intermediates and colonizes the embryonic tissue. The latter wave of EMPs buds from the yolk sac endothelium at E8.25 colonizes the liver after the establishment of embryo circulation, expands into monocytes, and differentiates into macrophages in tissues [69]. In brief, it has been confirmed that both embryo- and periphery-derived macrophages constitute the TAM pool in tumor tissues (Fig. 3).

Embryo-derived TAMs exhibit distinct phenotypes and divergent functions. For example, in a pancreatic ductal adenocarcinoma (PDAC) model, embryo-derived TAMs expressed significantly higher levels of CX3CR1 and lower levels of CD11a and CD49d. Moreover, self-renewing fetal-derived TRMs, as more potent drivers of PDAC progression, exhibited a distinct pro-fibrotic transcriptional profile compared with monocyte-derived TAMs and expanded through in situ proliferation during tumor progression [70]. In the omentum, embryonic CD163^+^Tim4^+^ TRMs exhibit a unique transcriptional profile and have been reported to play a causative role in ovarian cancer metastasis [9]. Besides, spatial interactions between TRMs and tumor cells promote tumor invasiveness and induce a potent Treg cell response for immune evasion [71] (Fig. 4). Studies have also confirmed that the depletion of TRMs significantly inhibits tumor progression [70]. Collectively, these data suggest that the embryonic origin of TAMs may differentially affect tumor progression.

Further studies are needed to determine the molecular markers of embryo-derived TAMs and their roles in tumor progression. A deeper understanding of embryo-derived TAMs will enable the identification of different targets for selective targeting to activate antitumor immunity.

## Technologies used for studying heterogeneous TAMCs

The technologies used to study the origin, chemotaxis, regulation, and function of the heterogeneous TAMCs are shown in Table 2.

**Table 2:** Technologies used for studying TAMCs

TAMCs	Contents	Technologies
TAM	Origin	Ms4a3^TdT^, Ms4a3^Cre^, Ms4a3^CreERT2^ mouse models to specifically dissect monocyte-differentiation pathways
	Biomarkers	F4/80, CD11b, CD86, CD206, etc
	Chemotaxis	Anti-CCL2 antibody, CCL5-KO mouse, etc., used to block TAM recruitment
	Regulation	Anti-M-CSF antibody, anti-CSF-1R antibody, etc., used to interfere with TAM differentiation
	Function	Liposomal clodronate, CD11b-DTR mouse, etc., used to verify the effect of TAM clearance
MDSC	Origin	Splenectomy is performed to determine the origin of splenic MDSC
	Biomarkers	CD11b, CD14, CD15, CD66b, HLA-DR (human) and CD11b, Ly6G, Ly6C (mouse), etc
	Chemotaxis	CXCR2 blockade, CXCR4 blockade, etc., used to inhibit MDSC recruitment
	Regulation	GM-CSF overexpression cell line (e.g. B16-GM), anti-GM-CSF antibody, etc., used to explore the factors regulating MDSC differentiation
	Function	(a) Anti-Gr-1 antibody, etc., used to consume MDSC (b) DR5 agonist, LXR agonist, etc., used to induce apoptosis of MDSC
TAN	Origin	Label by ^mNP^PFC (mNP specifically bind to CD177) to follow TAN
	Biomarkers	CD15, CD16, CD66b, CD14, HLA-DR (human) and CD11b, Ly6G (mouse), etc
	Chemotaxis	CXCR2 blockade, etc., used to inhibit TAN recruitment
	Regulation	Anti-TGF-βantibody, anti-IFN-γantibody, etc., used to explore the regulation of TAN polarization
	Function	(a) Clear by anti-Ly6G antibody (b) Induce by G-CSF
TADC	Origin	Zbtb46-GFP mouse to track TADC
	Biomarkers	BDCA-1, BDCA-2, BDCA-3, BDCA-4, CD141, CD1c, CD11c, IL-3Ra, CD45R, CD317, Siglec-H, etc
	Chemotaxis	Anti-CCL5 antibody, anti-CXCL12 antibody, etc., used to block TADC recruitment
	Regulation	Anti-TGF-β antibody, anti-TPO antibody, etc., used to explore the factors regulating TADC differentiation
	Function	CD11c-DTR mouse, Zbtb46-2A-CreERT2 mouse, etc., used to conditionally remove TADC

Ms4a3 is the specific gene expressed by GMPs

*mNP* murine neutrophil-specific peptide, *PFC* perfluorocarbon, *Zbtb46* (zinc finger and BTB domain-containing protein 46) is a transcription factor found in cDC, *GFP* green fluorescent protein

## Therapeutic strategies based on TAMCs

**Fig. 5:**
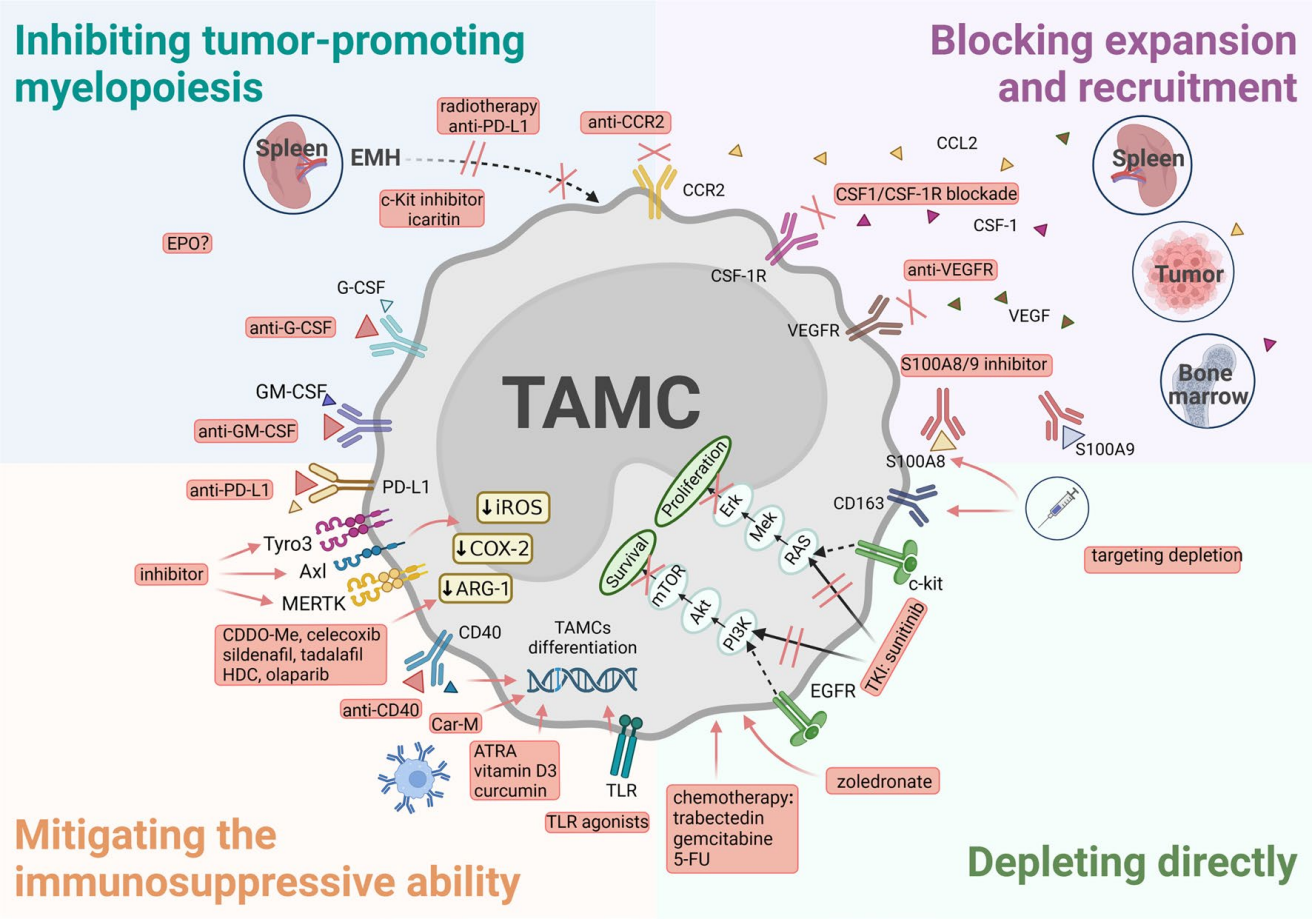
Potential therapeutic strategies for heterogeneous TAMCs. (1) Inhibiting tumor-promoting myelopoiesis. Intervention strategies aganist G-CSF and GM-CSF can reverse the direction of differentiation. Selectively eliminating tumor-promoting spleen EMH can inhibit the extramedullary TAMCs generation. The function of the EPO requires further discussion. (2) Blocking expansion and recruitment of TAMCs. Inhibition of CCL2/CCR2, VEGF/VEGFR and CSF-1/CSF-1R signaling pathways, and S100A8/9 can prevent the recruitment and accumulation of TAMCs. (3) Mitigating the immunosuppressive ability of TAMCs. In addition to PD-L1 inhibitor, inhibition of several myeloid receptor tyrosine kinases, and CDDO-Me, celecoxib, etc., can also mitigate the immunosuppressive ability of TAMCs. TLR agonists, anti-CD40 mAb, Car-M, and several small molecules can promote the maturation of TAMs and MDSCs. (4) Depleting directly. Zoledronate, sunitinib and some chemotherapeutic drugs can directly deplete TAMCs. Targeting scavenging receptor CD163 and S100A family proteins can selectively deplete TAMCs

Based on the diverse origins of TAMCs and their specific functional properties, several therapeutic strategies have been developed for antitumor immunotherapy. Herein, we propose four major therapeutic strategies (Fig. 5): (1) inhibiting tumor-promoting myelopoiesis, (2) blocking the expansion and recruitment of TAMCs, (3) mitigating immunosuppressive ability, and (4) depleting TAMCs directly. These strategies may provide feasible directions for targeting TAMCs of origin heterogeneity, and several preclinical studies and clinical trials have reported promising results.

### Inhibiting tumor-promoting myelopoiesis

#### Anti-G-CSF and anti-GM-CSF antibody

G-CSF and GM-CSF are essential for various generations of TAMCs, contributing to the myeloid-biased differentiation of BM and splenic HSPCs, trans-differentiation of erythroid precursors into myeloid cells, and induction of A2A receptor expression to promote TAM self-proliferation. Many studies have shown that intervention strategies targeting GM-CSF can reverse the differentiation direction and restore the antitumor function of TAMCs of diverse origins, which has great potential to enhance the efficacy of antitumor immunotherapy. For example, the combination of GM-CSF signaling blockade and gemcitabine suppressed MDSC generation and reversed T-cell inhibition in pancreatic cancer [72]. In addition, anti-GM-CSF antibodies can enhance the efficacy of tumor-specific adoptive T-cell therapy as well as combination therapy with anti-PD-1 and anti-CTLA4 antibodies [73]. Moreover, the crucial role of anti-G-CSF antibodies in promoting protective antitumor immunity has been demonstrated. Therefore, the combination of anti-G-CSF antibodies has important prospects for improving immunotherapy [74].

#### Targeting EMH

Notably, splenic EMH is a common upstream link between splenic HSPCs accumulation and the mass production of erythroid progenitor cells. Therefore, selective abrogation of tumor-promoting splenic EMH can inhibit EMH-triggered extramedullary myelopoiesis, which is particularly promising for restoring antitumor ability. For example, a low-dose c-Kit inhibitor can act on splenic HSPCs and reduce the expression of endogenous GM-CSF, thereby synergistically enhancing ICB efficacy [54]. Moreover, the CCL2/CCR2 signaling pathway plays a key role in selectively recruiting splenic HSPCs and triggering EMH-mediated myelopoiesis. Targeting CCL2/CCR2 may also be a powerful measure for inhibiting tumor-promoting myelopoiesis [54]. Other studies have shown that icaritin decreases the tumor infiltration of MDSCs and their immunosuppressive activity by blocking tumor-induced splenic myeloid-biased hematopoiesis in tumor-bearing mice, resulting in reduced tumor progression [75]. Hou et al. [76] reported that both local tumor-ionizing radiation and anti-PD-L1 treatment significantly decreased tumor-induced EPC abundance in the mouse spleen in an interferon- and CD8^+^ T cell-dependent manner.

#### EPO

Erythropoietin (EPO), an important indicator and trigger of EMH, is commonly used to treat anemia. Although it promotes red blood cell growth and differentiation, it does not improve the survival of patients with cancer or anemia. Studies have reported that Recombinant EPO promotes resistance to radiotherapy and anti-PD-L1 therapies by restoring the number of EPCs [76]. More importantly, EphB4, an EPO receptor, promotes tumor growth and progression [77]. Treatment with EPO-neutralizing antibodies has been found to significantly reduce tumor growth in B16-F10-bearing mice [78]. Therefore, the functions of EPO require further investigation.

### Blocking the expansion and recruitment of TAMCs

The CCL2/CCR2 signaling pathway contributes to tumor-promoting myelogenesis by enhancing splenic EMH and substantially promotes the migration and accumulation of MDSCs in tumor tissues. Studies have shown that immunosuppressive effects can be abrogated by anti-CCR2 antibody treatment to improve radiotherapy [79]. A Phase Ib study (NCT01413022) reported that patients with pancreatic ductal adenocarcinoma treated with the CCR2 inhibitor PF-04136309 combined with the chemotherapeutic regimen FOLFIRINOX showed a favorable response (Table 3) [80]. Blocking this pathway in combination with ICB therapy has yielded encouraging results. Compared to monotherapy, the CCR2 antagonist CCX872 in combination with anti-PD-1 therapy, can enhance the activation and reduce the exhaustion of intratumoral T cells, resulting in the slow progression of gliomas and a significant, durable survival advantage [81]. Moreover, the co-inhibition or dual blockade of CCL2 and PD-L1 can overcome the intrinsic resistance to PD-1/PD-L1 inhibition, which is of great significance for improving the clinical efficacy of ICB [82].

**Table 3:** Ongoing clinical trials targeting TAMCs

Strategies	Class	Treatment	Conditions	With ICBs or not	Outcomes	Adverse effects
Blocking expansion and recruitment of TAMCs	CCR2 inhibitor	PF-04136309, MLN1202, BMS-813160, etc	Pancreatic ductal adenocarcinoma, melanoma, metastatic cancer, etc	Both monotherapy and synergistic ICBs are available	ORR was 16.7–49%. About 33% of patients had reduced metastasis markers. But some trials have been stopped for lack of clinical benefit	Fatigue and anemia were common. DLTs were present. Neutropenia may occur with concurrent chemotherapy
	VEGFR inhibitor	Axitinib, Cabozantinib, Pazopanib, etc	Renal cell carcinoma, breast cancer, small cell lung cancer, etc	Both monotherapy and synergistic ICBs are available	OS and PFS were significantly prolonged, and ORR was increased	The incidence rate of serious AEs was about 40%. The most common AE was diarrhea
	CSF1R inhibitor	Cabiralizumab, PLX3397, ARRY-382, etc	Advanced solid tumor, melanoma, non-small cell lung cancer, etc	Mostly in collaboration with ICBs	The objective response rate was 0–16.7%. PFS did not prolong significantly	The incidence rate of serious AEs and DLTs was 0–54.5%. Gastrointestinal diseases were common
Mitigating the immunosuppressive ability of TAMCs	COX-2 inhibitor	Celecoxib, apricoxib, etc	Breast cancer, non-small cell lung cancer, etc	Collaboration with ICBs is rare	PFS did not significantly prolong or even shorten. There was no clinical benefit	The incidence rate of serious AEs was similar to that of placebo group
	PDE5 inhibitor	Tadalafil, Sildenafil, etc	Head and neck squamous cell carcinoma, prostatic neoplasms, etc	No	The activity of Arg-1 and iNOS was significantly decreased, reversing tumor specific immune suppression	No AEs are reported
	TLR agonist	CMP-001, Imiquimod, SD-101, etc	Melanoma, head and neck cancer, breast cancer, etc	Mostly in collaboration with ICBs	ORR was 0–55.6%, and progressive disease accounted for 11.1–44.4%. MPR also showed a mix of good and bad outcomes	The incidence rate of serious AEs was 16.67–44.4%. Chills and fatigue were prominent
Direct depletion	VEGFR inhibitor	Sunitinib malate, ZD6474, Apatinib, etc	Renal cell carcinoma, bladder cancer, breast cancer, gastric cancer, etc	Both monotherapy and synergistic ICBs are available	PFS and OS were prolonged in most trials. Some studies have shown that MDSC mediated immunosuppression is reversed	The incidence rate of serious AEs was 6.67–40%. Leukocytes and platelets were often affected
	C-kit inhibitor	Imatinib, Masitinib, Dasatinib, Dovitinib, etc	Salivary gland neoplasm, non-small cell lung cancer, melanoma, thyroid cancer, etc	Collaboration with ICBs is rare	Clinical benefit was limited. PFS was prolonged in some trials, but the results of some trials were mainly progressive disease	The incidence rate of serious AEs was 2.44–65%. Among them, diarrhea, nausea and vomiting were easy to occur
	Chemo therapy	Cisplatin, 5-FU, Carboplatin, Paclitaxel, Doxorubicin, etc	Esophageal neoplasms, breast cancer, non-small cell lung cancer, ovarian cancer, sarcoma, etc	Both monotherapy and synergistic ICBs are available	Effectiveness varies greatly depending on tumor type, drug dose, combination therapy, etc. Both trials with significant clinical benefit and trials with low ORR exist	Nausea, constipation and diarrhea were prominent in AEs. The hematopoietic system such as neutrophils and platelets were susceptible

*ORR* objective response rate, *DLT* dose limiting toxicities, *OS* overall survival, *PFS* progression-free survival, *AE* adverse event, *MPR* major pathologic response

In addition to CCL2/CCR2, the VEGF/VEGFR signaling pathways can be successfully targeted in cancer to prevent TAMCs recruitment and accumulation [83, 84]. More interestingly, targeting VEGF/VEGFR reduced the intratumoral infiltration of TAMCs and improved ICB's efficacy (Table 3). For example, selective VEGFR-1 inhibition enhances the efficacy of anti-CTLA-4 and anti-PD-1 mAbs [85]. The CSF-1/CSF-1R signaling pathway is also a primary target for inhibiting MDSC recruitment to tumor sites to constrain tumor progression. In preclinical tumor models, improved effects were observed when CSF1/CSF-1R blockade was combined with irradiation, paclitaxel, anti-VEGFR antibody, and ICB [86, 87]. Additionally, CSF-1R inhibition and CXCR2 antagonism have been combined to reduce TAM and PMN-MDSC populations and improve anti-PD-1 efficacy [88].

### Mitigating the immunosuppressive ability of TAMCs

#### Common approaches

A common approach to restoring antitumor immune function, regardless of TAMCs origin, is to mitigate their immunosuppressive ability. Recent studies have found that the nuclear factor erythroid 2-related factor 2 (Nrf2) pathway activator CDDO-Me reduces intracellular ROS production, decreasing tumor growth [89]. Moreover, the COX-2/PGE2/EP axis maintains suppressive functions of myeloid cells, such as ARG-1 expression [20, 90, 91]. Therefore, therapies targeting COX-2, such as celecoxib, are of great interest, and the relevant clinical trials are shown in Table 3 [92]. In preclinical mouse models, PDE-5 inhibitors, such as sildenafil and tadalafil, abrogate the immunosuppressive mechanisms of MDSCs by downregulating iNOS and ARG-1 activities [93–95]. Clinical trials have also shown promising antitumor effects in patients with head and neck squamous cell carcinoma and metastatic melanoma (Table 3) [96, 97].

However, reducing the inhibitory power of TAMC alone is unlikely to completely eliminate tumors, and its combination with ICB therapy is a promising cancer treatment strategy. For example, the antitumor mechanism of histamine dihydrochloride (HDC) involves a decrease in the ability of MDSC to inhibit CD8^+^ T cell proliferation by reducing the formation of NOX2-derived ROS. HDC was particularly effective in combination with PD-1/PD-L1 inhibition compared to PD-1/PD-L1 inhibition alone [98]. In addition, olaparib at sub-IC50 concentrations can block the expression of ARG-1, iNOS, and COX-2 and reduce the inhibitory function of MDSC, which is more effective in blocking tumor progression when used in conjunction with anti-PD-1 [99]. Remarkably, several promising pharmacological targets exist, such as the myeloid receptor tyrosine kinases TYRO3, AXL, and MERTK. Studies have confirmed that inhibition of these targets can diminish the suppressive ability of MDSC, slow the growth of tumors, and increase the infiltration of CD8^+^ T cells. In particular, the inhibition of these targets has been shown to augment the efficacy of anti-PD-1 immunotherapy, offering promising applications [100].

Regaining the antitumor ability of TAMCs is more radical than simply reducing their immunosuppressive ability. For example, TLR agonists efficiently skew TAM from an M2-like state to an M1-like state, which has been evaluated in many clinical trials (Table 3) [101, 102]. The TLR7/8 agonists 3M-052 and NKTR-262, in combination with PD-1 blockade, can prolong therapeutic efficacy and duration [103]. Anti-CD40 mAbs promote TAM transformation to the antitumor phenotype [104], and synergistic treatment with anti-CSF-1R antibodies stimulates the antitumor activities of TAMs and assists tumor remission [105]. Additionally, several small molecules, such as ATRA, vitamin D3, and curcumin, can induce the differentiation of MDSCs and reestablish tumoricidal immunity [106–110]. Interestingly, studies have shown that the blockade of PD-1-mediated signals, as a representative ICB therapy, can diminish the accumulation of immunosuppressive and tumor-promoting MDSCs, skewing the myeloid lineage fate to mature myeloid effector cells [111].

#### Chimeric antigen receptor macrophage (Car-M)

Car-M therapy, in which macrophages are modified with a specific chimeric antigen receptor (CAR), converts the TAM from M2 to M1. With its antigen-specific phagocytosis and tumor clearance abilities, Car-M can easily invade the TME and enhance the antitumor activity of T cells. Therefore, compared with CAR-T therapy, which is difficult to use in the TME, CAR-M therapy has shown great potential for treating solid tumors such as ovarian cancer and has entered clinical evaluation [112]. However, CAR-M therapy has several limitations, including possible cytokine release syndrome and weak antitumor ability in vivo. Therefore, further studies are needed to fully elucidate the structure of CAR and the mechanism of CAR-M therapy.

In addition, one of the serious obstacles to the clinical application of CAR-related therapies is their high manufacturing cost. In this context, the recently proposed universal CAR (UniCAR), which targets different epitopes in a modular design, can not only significantly reduce the cost of CAR-T therapy but also avoid antigen escape and use in relapsed or refractory cancers [113]. This research progress is of great value for the clinical application of Car-M therapy and may be a direction worth exploring in the future.

### Direct depletion

Several targeting strategies have been developed based on TAMCs depletion to directly combat their negative effects. For instance, the tyrosine kinase inhibitor sunitinib has been successfully used to deplete MDSCs in patients with renal cell carcinoma by blocking VEGF and c-kit signaling, which are involved in the generation of MDSCs (Table 3) [36, 114, 115]. Moreover, chemotherapy is effective in eliminating TAMCs. The antitumor activity of trabectedin relies on the depletion of TAMs via the induction of apoptosis [116]. In a clinical trial, gemcitabine treatment of patients with pancreatic cancer resulted in a dramatic decrease in PMN-MDSCs [117]. In addition, gemcitabine/5-FU can reduce immunosuppression by depleting MDSCs and increasing the intratumoral infiltration of CD4^+^ and CD8^+^ T cells [118]. One clinical trial (NCT03189719) reported that cisplatin/5-FU combined with a PD-1 inhibitor showed promising efficacy (Table 3). Zoledronate has been shown to inhibit tumor progression by inducing TAM apoptosis and reducing TAM infiltration in different preclinical tumor models [119–122]. However, this strategy can lead to the loss of beneficial macrophages. Thus, more selective strategies are being developed, such as targeting the scavenging receptor CD163 to selectively deplete M2-like TAMs and targeting S100A family proteins to selectively deplete TAMCs [123, 124]. An important direction for future research on TAMCs of diverse origins is to identify additional biomarkers that can aid in developing specific intervention strategies for their elimination.

## Conclusions

The limited effectiveness of clinical interventions targeting TAMCs in the field of cancer immunotherapy can be attributed to the incomplete understanding of the heterogeneity of TAMCs. In this review, we provide a comprehensive summary of the mainstream views of the heterogeneity of TAMCs. However, high-resolution analytical methods such as single-cell techniques continue to improve our understanding of heterogeneity. For complex high-throughput data, some studies have constructed specific signaling maps of different cell types, such as macrophages, dendritic cells, and myeloid suppressor cells, revealing anti- or pro-tumor subpopulations, supporting TAMC functional data visualization [125]. Therefore, with continuous technological changes, the heterogeneity of TAMCs will develop toward functionality and refinement. At present, the discussion of TAMCs heterogeneity still focuses on subgroups, such as M1 and M2, M-MDSCs and PMN-MDSCs, N1 and N2, pDC, cDC, and moDCs, while ignoring the origin as an important part of heterogeneity. Therefore, the current torsion strategy remains effective against classical BM-derived TAMCs. In addition, the notion that TAMCs are solely classified into antitumor and pro-tumor types is insufficient to capture the extensive functional diversity of TAMCs. Currently, research on EMH is still in its infancy, and improving tumor immunotherapy from the perspective of origin heterogeneity is a new topic.

Targeting TAMCs of heterogeneous origin to limit the tumor-promoting myeloid response is an attractive strategy, given that the origin may drive intrinsic phenotypic and functional differences in TAMC as well as varying effects on tumor progression. In addition to the classical origin of myeloid-biased differentiation in the BM, advances have shown that the origins of TAMCs include the abnormal differentiation of splenic HSPCs, erythroid progenitor cells, and B precursor cells in the spleen and embryo-derived TAMCs in local tumors. More importantly, these non-classical TAMCs differ in their immunosuppressive function when educated and shaped in the TME, which greatly impacts ICB therapy's antitumor efficacy. Therefore, targeting TAMCs should not be limited to classical TAMCs derived from the BM but must consider the complex regulatory mechanisms of origin and diverse phenotypic characteristics of TAMCs. Targeted intervention strategies for TAMCs of heterogeneous origin, such as inhibiting tumor-promoting myelopoiesis, blocking the expansion and recruitment of TAMCs, mitigating immunosuppressive ability, and depleting TAMCs directly, can overcome immunosuppression in the TME and enhance the antitumor efficacy of ICB synergistic therapy, which has great clinical prospects.

## Future perspectives

At present, the heterogeneous composition, and the regulatory mechanism of origin and function of TAMCs are not fully understood, the challenges in the future study of TAMCs are mainly as follows: (1) beyond the above, it is unclear whether TAMCs has other sources, and is an excellent entry point for expanding the heterogeneity of TAMCs in the future; (2) the functions of heterogeneous TAMCs are not fully understood, so it is necessary to reveal their independent effects and interactions with other immune cells; (3) the phenotypes of non-classical TAMCs are not well defined, and more biomarkers need to be identified to distinguish different types of TAMCs for better identification, tracking, and intervention; (4) the regulation mechanism of TAMCs needs to be further studied, and new molecules that promote the acquisition of inhibitory phenotypes of TAMCs may still remain unidentified; (5) optimizing TAMCs targeting strategies from the perspective of origin and combining with ICBs to obtain better efficacy are both important directions for the development of tumor immunotherapy, which are worthy of further exploration; and (6) the above studies were mostly confirmed in animals, and in the future, extensive human data are needed to demonstrate the clinical translational feasibility of studies on TAMCs.

In conclusion, TAMCs of heterogeneous origin show great promise for tumor immunotherapy. However, more in-depth investigations are required to remodel the TME by targeting TAMCs, thereby improving ICB efficacy.

## Abbreviations

AMP: Adenosine monophosphate
ACEi: Angiotensin converting enzyme inhibitor
AGTR1: Angiotensin II type 1 receptor
BM: Bone marrow
Car-M: Chimeric antigen receptor macrophage
CAR: Chimeric antigen receptor
CLP: Common lymphoid progenitor
CMP: Common myeloid progenitor
COX2: Cyclooxygenase-2
CSF-1: Colony-stimulating factor 1
EDMCs: Erythroid-differentiated myeloid cells
EMH: Extramedullary hematopoiesis
EMPs: Erythro-myeloid progenitors
EPCs: Erythroid precursor cells
EPO: Erythropoietin
GMPs: Granulocyte–macrophage progenitors
GM-CSF: Granulocyte–macrophage colony-stimulating factor
G-CSF: Granulocyte colony stimulating factor
HCC: Hepatocellular carcinoma
HDAC-2: Histone deacetylase 2
HDC: Histamine dihydrochloride
HSPCs: Hematopoietic stem and progenitor cells
ICB: Immune checkpoint blockade
iNOS: inducible nitric oxide synthase
IDO: Indoleamine 2, 3-dioxygenase
MMP: Matrix metal protein
M-CSF: Macrophage colony-stimulating factor
MEPs: Megakaryocyte-erythroid progenitors
MDSC: Myeloid-derived suppressor cell
NK: Natural killer TregsRegulatory T-cells
Nrf2: Nuclear factor erythroid 2-related factor 2
OS: Overall survival
PPARγ: Proliferator receptor gamma
Pre-B: B-cell precursors
PDAC: Pancreatic ductal adenocarcinoma
ROS: Reactive oxygen species
TAN: Tumor associated neutrophil
TAM: Tumor associated macrophage
TADC: Tumor associated dendritic cell
TAMCs: Tumor-associated myeloid cells
TGF-β: Transforming growth factor-beta
TME: Tumor microenvironment
TNF-α: Tumor necrosis factor-alpha
TRMs: Tissue-resident macrophages
VEGF: Vascular endothelial growth factor
